# Tuberculosis among Patients Admitted to the Department of Medicine of a Tertiary Care Center in Nepal: A Descriptive Cross-sectional Study

**DOI:** 10.31729/jnma.5938

**Published:** 2021-06-30

**Authors:** Suman Thapa, Anupam Bista, Prashant Subedi, Aaradhana Adhikari, Sunil Pokharel

**Affiliations:** 1 Department of Medicine, Patan Hospital, Patan Academy of Health Sciences, Lalitpur Nepal; 2 Center for Tropical Medicine and Global Health, Nuffield Department of Clinical Medicine, University of Oxford, Oxford, United Kingdom.

**Keywords:** *clinical profile*, *risk factors*, *tuberculosis*

## Abstract

**Introduction:**

Tuberculosis has high burden in developing countries like Nepal. This study aims to determine the prevalence of tuberculosis among patients admitted in the department of medicine of a tertiary hospital.

**Methods:**

A descriptive cross-sectional study of all the patients admitted to the tertiary care hospital from 1^st^ January 2017 to 31^st^ December 2019 was done. Ethical approval was obtained from Institutional Review Committee (Ref: drs2006181387). Convenience sampling method was used. A descriptive analysis of demographic, clinical and laboratory profile of patients was made using Microsoft Excel version 2016. Point estimate at 95% Confidence Interval was calculated along with frequency and proportion for binary data.

**Results:**

Among 6829 patients admitted to the department of medicine, 209 (3.06%) (2.65-3.47 at 95% CI) patients were diagnosed with tuberculosis. Among them, 147 (70.33%) were males and the mean age was 49.77 years. Pulmonary and extra-pulmonary tuberculosis were present in 153 (73.20%) and 56 (26.79%) patients, respectively. Bacteriological confirmation was limited to 107 (70%) of pulmonary tuberculosis and 3 (5%) of extrapulmonary tuberculosis. Fever was the commonest presentation in 166 (79.42%) followed by cough in 164 (78.46%), anorexia in 108 (51.67%), weight loss 104 (49.76%), and others.

**Conclusions:**

The study showed that the prevalence of tuberculosis among admitted patients was higher than national prevalence.

## INTRODUCTION

Tuberculosis is one of the commonest causes of morbidity and mortality worldwide.^1,2^ Evolving resistance to anti-tubercular drugs is a huge therapeutic challenge.^3^ In Nepal, around 69,000 people develop new active infections every year.^4^ Human immunodeficiency Viruses (HIV) co-infection, smoking and diabetes are significantly associated with tuberculosis infection and attributable deaths.^5-7^ Tuberculosis commonly presents with cough, fever, hemoptysis, anorexia and unintentional weight loss.^5^ Presentation varies depending upon infection site and other patient factors.^8^ A non-specific presentation, limited utility of culture, suboptimal sensitivity of acid fast bacilli (AFB) stain^9^ and unavailability of test like Xpert Mycobacterium tuberculosis (MTB)/rifampicin (RIF) at the point-of-care^12,13^ test make diagnosis a challenge.

Attempts to understand tuberculosis from the clinical and laboratory perspective in Nepal have largely been focused on outpatients.^10^ Hospitalized patients are characterized differently and need special attention for early diagnosis and management.^11^

This study aims to determine the prevalence of tuberculosis among patients admitted in the department of medicine of a tertiary hospital.

## METHODS

A descriptive cross-sectional study of patients admitted to the Department of Medicine of Patan Hospital from 1^st^ January 2017 to 31^st^ December 2019 was done. Ethical approval was obtained from Institutional Review Committee-Patan Academy of Health Sciences (Ref: drs2006181387) before the start of data collection. Convenience sampling was done and the sample size was calculated as,

$$\begin{array}{ll} \text{n} & \text{= }{\text{Z}}^{\text{2}}\times \text{p}\times \text{(1-p)}/{\text{e}}^{\text{2}}\\ & \text{= }{\left(1.96\right)}^{\text{2}}\times (0.5)\times (1-0.5)/{(\text{0.02)}}^{\text{2}}\\ & \text{= }2401 \end{array}$$

Where,

n = minimum required sample size
Z = 1.96 at 95% Confidence Interval (CI)
p = prevalence taken as 50% for maximum sample size
q = 1-p
e = margin of error, 2%

As convenience sampling technique was used, the sample size was doubled to 4802. Adding 10% for missing data, the sample size of 5282 was reached. But we collected data from 6829 patients.

All adult patient (15 years of age and above) admitted in medical ward, geriatric ward, step down, medical intensive care unit and private wards under Medicine Department of Patan hospital with diagnosis of tuberculosis (both pulmonary and extra pulmonary) from 1st January 2017 to 31st December 2019 were included in the study. A total of 209 hospital records of patients with tuberculosis were identified and analyzed.

We used the Centers for Disease Control and Prevention (CDC) definitions for smoking.^12^ Those patients who had never smoked or who had smoked less than 100 cigarettes in their lifetimes were defined as never smokers. Those patients who had smoked at least 100 cigarettes in their lifetimes but had quit smoking at time of interview were former smokers. Others who smoked currently at the time of interview and had smoked more than 100 cigarettes in lifetime were current smoker. The amount of smoking was expressed as pack year which was calculated by multiplying the number of packs of cigarettes smoked per day by the number of years the person had smoked.

A known case of HIV as per previous report or patients' self-declaration was considered as positive for HIV. For patients with unknown HIV status, case file and investigation were reviewed and were grouped as positive or negative based on available information. Those patients whose HIV reports were not available were grouped as status unknown.

A diagnosis of patients status for diabetes was established based on patients history, self-declaration, or report as available in case files. Blood investigation criteria for diagnosis of diabetes included fasting plasma glucose of more than 125mg/dl, two-hour postprandial plasma glucose level more than 199 mg/ dl or glycated hemoglobin (HbA1C) more than 6.4%.^13^

Anemia was said to be present when hemoglobin level was below 12 gm/dl in female and 13 gm/dl in male. Anemia was further classified into mild, moderate and severe using the blood hemoglobin level cut-offs of 11 gm/dl and above, 8-10.9 gm/dl and below 8 gm/dl respectively.^14^

Hospital records of patients admitted with a diagnosis of tuberculosis were identified from the patient record section of the hospital, which archives the patient records following International Statistical Classification of Diseases and Related Health Problems Version 10.^15^ Individual patient files were accessed and reviewed for relevant data by the study investigators. A standard performa was used to retrieve and record the demographic, clinical and laboratory data of the patients. The proforma was anonymized generating a unique patient identifier for each patient.

Data was collected in standard paper proforma and later entered into a secure computer system using Microsoft Excel Version 2016. A descriptive analysis of all obtained data was done and point estimate at 95% CI was calculated along with frequency and proportion for binary data.

## RESULTS

Among 6829 patients admitted in department of medicine of Patan Hospital, 209 (3.06%) (2.65-3.47 at 95% CI) were diagnosed with tuberculosis. Out of the 209 patients diagnosed with tuberculosis, 147 (70.33%) were males and 62 (29.67%) were females, and the mean age of participants was 49.77 years. The mean length of hospital stay among these patients was 6.9 days.

**Figure 1:**
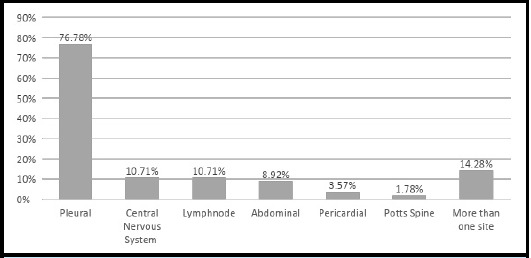
Sites of extrapulmonary tuberculosis infection (n = 56).

153 (73.20%) patients had pulmonary tuberculosis, with involvement of at least one extrapulmonary sites in 29 (18.95%) patients and more than one in 6 (3.92%) patients. 56 (26.79%) patients had extrapulmonary tuberculosis with involvement of various organs (Table 1, Figure 1).

**Table 1:** Pulmonary Tuberculosis with extrapulmonary dissemination.

Extrapulmonary dissemination in patients with pulmonary tuberculosis	n (%)
Pleural	10 (6.53)
Central Nervous System	6 (3.92)
Abdominal	11 (7.18)
Potts's spine	2 (1.30)
Lymph node	5 (3.26)
Osteomyelitis	1 (0.65)
Total	35 (100)

Smoking, diabetes, and HIV co-infection was present in 90 (43.06%), 25 (11.96%) and 3 (1.43%) patients, respectively. 64 (71.11%) of the smokers were current smokers. The mean pack years among 87 smokers was 15.21. Mean HbA1c among 23 diabetic patient was 8.65%.

**Figure 2:**
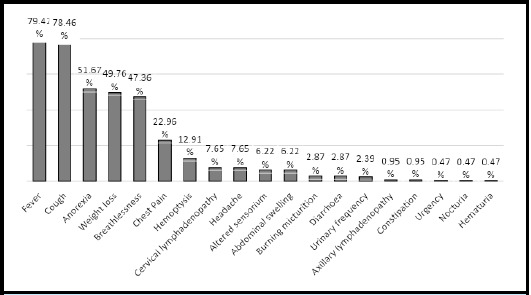
Clinical profile of patients with tuberculosis (n = 209).

Fever was the commonest presentation in 166 (79.42%) followed by cough in 164 (78.46%), anorexia in 108 (51.67%), weight loss 104 (49.76%). Other symptoms were breathlessness in 99 (47.36%), chest pain in 48 (22.96%), haemoptysis in 27 (12.91%) patients (Figure 2).

Laboratory investigations showed the abnormal leukocyte counts in 68 (32.53%) patients, raised neutrophil lymphocyte ratio (NLR) in 167 (79.91%) patients, raised erythrocyte sedimentation rate (ESR) in 119 (92.25%) patients and anemia in 177 (84.69%) patients (Table 2).

**Table 2:** Laboratory profile of patients with tuberculosis.

Laboratory parameters	n (%)
Abnormal WBC count	68 (32.5)
Leukopenia	4 (1.91)
Leukocytosis	64 (30.62)
Increased NLR ratio ≥ 2.9	167 (79.9)
Raised ESR	119 (92.25)
20 -99 mm/hr	77 (59.69)
≥100 mm/hr	42 (32.55)
Anemia	177 (84.69)
Mild anemia	55 (26.31)
Moderate anemia	106 (50.72)
Severe anemia	16 (7.66)

Among all patients who underwent sputum microscopy, 73 (34.93%) patients had positive sputum smear for AFB. Data on sputum for Xpert MTB/RIF was available for 80 patients (14 positive for sputum AFB and 66 AFB negative patients), out which 48 (60%) were positive. 14 (100%) patients with AFB positive sputum and tested for Xpert MTB/RIF were positive for Xpert MTB/RIF. However, among 66 patients with sputum for AFB negative status, 34 (51.51%) patients were positive for Xpert MTB/RIF (Table 3).

**Table 3:** Positive diagnostic test results.

Variables	n (%)
Sputum AFB (n= 209)	73 (34.93)
Sputum Xpert MTB/RIF (n= 80)	48 (60)
Lymph node for AFB (n= 32)	1 (3.12)
Tissue Xpert MTB/RIF (cerebrospinal fluid, lymphnode) (n= 23)	2 (8.69)

Among 153 patients of pulmonary tuberculosis, 73 (47.71%) patients had sputum positive for AFB, 34 (22.22%) patients were positive by Xpert MTB/ RIF and 14 (9.15%) patients were positive for both AFB and Xpert MTB/RIF tests. In total, 107 (69.93%) patients were bacteriologically confirmed pulmonary tuberculosis whereas 46 (30.07%) were clinically diagnosed pulmonary tuberculosis.

Among 56 patients of extra pulmonary tuberculosis, only 3 (5.35%) had tissue sample positive for either AFB or Xpert MTB/RIF and the remaining 53 (94.65%) patients were clinically diagnosed with extra pulmonary tuberculosis.

## DISCUSSION

Tuberculosis has high burden in Nepal. National tuberculosis prevalence survey 2018-19 has estimated the prevalence of tuberculosis to be 0.41% among the population.^16^ Three percent patients among all admissions in the medicine department in the present study were diagnosed with tuberculosis. Although relatively lower prevalence of tuberculosis is observed in hospital settings as a large proportion of patients are managed in outpatient basis,^7^ hospitalized patients imply more severe infections and/or presence of coinfections and thus requires special consideration for the diagnosis and management.

Tuberculosis can infect anyone irrespective of their age and sex but is common in young males, the commonest age group being 25-34 years and male to female ratio 1.7:1 reported previously.^15^ The older population commonly infected in the present study can largely be attributed to high risks of multiple co-morbidities and severe tuberculosis infection requiring hospital admission in older patients.^11^ Pulmonary tuberculosis is the commonest site of tuberculosis infection accounting for more than 80% of all tuberculosis, followed by lymphatic system as the commonest extrapulmonary site.^5^ Previous studies from Nepal comprising outpatients and inpatients have shown high prevalence of lymph node involvement, contributing to up-to 42% of all extra-pulmonary tuberculosis.^16^ Higher involvement of pleura 77% and central nervous system 11% in the present study conducted among inpatients could be due to the high severity with involvement of these sites needing hospitalization. This finding supports the previous finding of pleural involvement (55% and 73%) as the commonest extrapulmonary site among hospitalized patients.^4,18^

Diagnosis of tuberculosis remains a major challenge in the developing countries.^19^ In 2018, only around half of all reported pulmonary tuberculosis globally were bacteriologically confirmed.^1^ Although, data from high income settings with easy access to sensitive diagnostic tests like Xpert MTB/RIF have reported higher rates of bacterial confirmation,^1^ timely diagnosis lags behind in the developing countries.^19^ The conventional AFB microscopy has limited sensitivity in diagnosis^9^ and Xpert MTB/RIF is not readily available in resource-poor settings.^20^ Bacteriological confirmation limited to 70% of pulmonary tuberculosis and 5% of extrapulmonary tuberculosis in the present study with on-site facilities for microscopy and Xpert MTB/RIF points to the limited functional capacity of test facilities and underutilization of available tests in clinical diagnosis.

Only 5-10% of people infected with Mycobacterium tuberculosis develops tuberculosis disease but the risk is significantly higher among people with HIV co-infection, smoking and diabetes.^5^ HIV co-infection increases the risk for tuberculosis infection by 30 folds^5^ and significantly increases the rate of progression of latent tuberculosis infection to disease.^21^ National Tuberculosis Program has emphasized for screening of HIV infection in all tuberculosis patients^7^ but the coverage of HIV testing in tuberculosis patients remain low in Nepal. HIV status was known in only around 40% patients in the present study with HIV prevalence of 1.43% among the population tested. This finding emphasizes on the need for strengthening HIV screening among all patients with tuberculosis.

Smoking has strong adverse effect on immune system of pulmonary tree and increases susceptibility to tuberculosis in a dose response manner.^22,23^ Smokers are twice as likely to be infected with tuberculosis and die from the disease.^5^ Similarly, Diabetes Mellitus also increases the risk of tuberculosis infection by two to three folds and has negative effect on treatment outcome.^5,24^ The high prevalence of smoking and diabetes in the present study supports the similar findings reported previously.^24^

Tuberculosis in most of the patients presents with clinical syndrome of respiratory symptoms like prolonged cough and hemoptysis, and non-specific constitutional symptoms like fever, loss of appetite and unintentional weight loss.^13,25^ The clinical presentation varies widely depending on the site of infection and other patient variables, but are often empirically used to make a diagnosis of tuberculosis in resource-poor settings with limited access to reliable tuberculosis diagnostics. A cautious approach to obtaining detailed clinical data combined with laboratory findings like blood counts^26-28^ and ESR^5^ in the absence of specific tuberculosis diagnostic can improve the diagnostic outcomes and guide monitoring of treatment response. Approaches to looking at relative neutrophil elevation compared to lymphocytes (neutrophil-lymphocyte ratio) is highly predictive of tuberculosis progression^26-28^ and should be routinely used in the monitoring of tuberculosis patients.

Given the retrospective nature of the analysis done on the secondary data of hospital records, there were limitations in the study. The possibility of missing data, especially the negative findings in the patient records could have influenced the results. However, attempts were made to review the complete patient records for pertinent data, and the findings from patients' history and laboratory records over the course of hospital admission were triangulated to retrieve the best available evidence.

## CONCLUSIONS

Tuberculosis is a common cause of infections among hospital admitted patients in Nepal. Although a classic presentation of cough and fever are generally expected in tuberculosis, a large proportion of hospital admitted patients have extra pulmonary involvement which often present with atypical symptoms. Conventional AFB microscopy has limited sensitivity for the diagnosis of tuberculosis and improved access to reliable point-of-care diagnostic tests like Xpert MTB/RIF is urgently required. Findings from detailed history taking and hematological and biochemistry testing should be cautiously interpreted for timely identification and treatment of tuberculosis.
